# Collective-Intelligence Recommender Systems: Advancing Computer Tailoring for Health Behavior Change Into the 21st Century

**DOI:** 10.2196/jmir.4448

**Published:** 2016-03-07

**Authors:** Rajani Shankar Sadasivam, Sarah L Cutrona, Rebecca L Kinney, Benjamin M Marlin, Kathleen M Mazor, Stephenie C Lemon, Thomas K Houston

**Affiliations:** ^1^ Division of Health Informatics and Implementation Science Department of Quantitative Health Science University of Massachusetts Medical School Worcester, MA United States; ^2^ Meyers Primary Care Institute University of Massachusetts Medical School Worcester, MA United States; ^3^ Division of General Medicine and Primary Care University of Massachusetts Medical School Worcester, MA United States; ^4^ College of Information and Computer Sciences , University of Massachusetts Amherst Amherst, MA United States; ^5^ Division of Preventive and Behavioral Medicine University of Massachusetts Medical School Worcester, MA United States; ^6^ eHealth Quality Enhancement Research Initiative (QUERI) Center for Healthcare Organization and Implementation Research (CHOIR) Veteran’s Health Administration Bedford, MA United States

**Keywords:** computer-tailored health communication, machine learning, recommender systems

## Abstract

**Background:**

What is the next frontier for computer-tailored health communication (CTHC) research? In current CTHC systems, study designers who have expertise in behavioral theory and mapping theory into CTHC systems select the variables and develop the rules that specify how the content should be tailored, based on their knowledge of the targeted population, the literature, and health behavior theories. In collective-intelligence recommender systems (hereafter recommender systems) used by Web 2.0 companies (eg, Netflix and Amazon), machine learning algorithms combine user profiles and continuous feedback ratings of content (from themselves and other users) to empirically tailor content. Augmenting current theory-based CTHC with empirical recommender systems could be evaluated as the next frontier for CTHC.

**Objective:**

The objective of our study was to uncover barriers and challenges to using recommender systems in health promotion.

**Methods:**

We conducted a focused literature review, interviewed subject experts (n=8), and synthesized the results.

**Results:**

We describe (1) limitations of current CTHC systems, (2) advantages of incorporating recommender systems to move CTHC forward, and (3) challenges to incorporating recommender systems into CTHC. Based on the evidence presented, we propose a future research agenda for CTHC systems.

**Conclusions:**

We promote discussion of ways to move CTHC into the 21st century by incorporation of recommender systems.

## Introduction

Are there aspects of the Web 2.0 phenomenon that can be marshaled by public health practitioners to improve community and individual health or advance scientific goals?

[1]

Theory-based, computer-tailored health communication (CTHC) is a tool that is frequently used to support behavior change [2]. It builds on the concepts of personal relevance, relatedness, and cultural similarity, which are constructs of multiple behavioral theories including the transtheoretical model, the theory of reasoned action, social cognitive theory, and self-determination theory [3-5]. Current CTHC systems use selected variables from patients’ baseline profile and if-then rules to send tailored messages to specific subsets of patients [2,6-10]. Study designers who have expertise in behavioral theory and mapping theory into CTHC systems select the variables and develop the rules that specify how the content should be tailored (what messages need to be sent to that patient subset) based on their knowledge of the targeted population, the literature, and health behavior theories. Textbox 1 provides an illustrative example of how a current CTHC system might tailor a message as part of a smoking cessation intervention. Over 30 years of research testing CTHC approaches have provided convincing evidence of the effectiveness of tailored messages [6,7,11-16]. Technological advances have enabled CTHC to be delivered on multiple platforms (eg, websites, email, and mobile) and to reach large populations. However, as CTHC systems are currently implemented, we may have reached the natural limits of their ability to tailor communications.

**Computer-tailored health communication (CTHC): a simple example of a tailored message addressing weight gain on a Web-assisted tobacco intervention.**

John Smith, a 38-year-old smoker, has been smoking for 15 years. He has made multiple quit attempts in the past, but during each attempt he gained between 10 and 20 pounds. Fear of weight gain is a significant barrier to another quit attempt.

John is trying to quit again and registers on the Decide2Quit.org Web-assisted tobacco intervention. For 8 weeks, the system sends 2 tailored emails per week to John to help him quit.

Current CTHC

- In this approach, tailoring is based on information that John provides when he registers. For this example, we focus on 1 characteristic only: gender.
- Since women are typically more concerned about weight gain after quitting [17-21], the developers of Decide2Quit.org have specified that half of the emails sent to women should contain information related to weight gain, but only a quarter of the emails sent to men should be focused on weight gain.
- After registering on Decide2Quit.org, John receives the first email that targets weight loss in the second week (third message) of the intervention. John likes the message and finds the tips it offers useful. He looks forward to receiving similar messages. However, the next 5 messages he receives focus on other topics. The next message with information on weight gain arrives only in week 5.
- John does not think the system helped and fails in his attempt to quit.

Recommender CTHC

- In this approach, the message is selected based on the collective-intelligence data, not on preset rules.
- After registering on Decide2Quit.org, John visits the weight loss page on the website (implicit data). The system uses these data and selects 1 of the messages targeting weight loss and sends it to John in week 2 (third message). John likes the messages and rates the message highly (explicit data). The system then notes both of these items of implicit and explicit feedback and regularly sends messages targeting weight gain to John. The system also repeats the message that John rates highly.
- Because the intervention targeted his needs more specifically, John finds these messages useful and succeeds in his attempt to quit.
- We have kept the example simple to be easily understandable. We have not included in this example how the group’s feedback can help John.

New approaches to tailoring based on collective intelligence may be able to build on the successes and lessons learned from past tailoring efforts, and may overcome the limitations inherent in current CTHC systems. Many people already encounter collective-intelligence tailoring as they interact with companies like Netflix and Amazon. These companies have developed a special class of machine learning algorithms (recommender systems) to tailor content. These systems tailor content based on collective-intelligence data (ie, data derived from the behavior of users as they interact with the system) in addition to user profiles [22-24].

Collective-intelligence data include implicit and explicit user feedback. Implicit data are derived from user actions (eg, the website view patterns of each individual accessing the system). Explicit data consist of self-reported item ratings (eg, ratings provided by users for items such as books or movies, often on a 5-star scale). However, in the health-promotion arena, patients could be asked to rate relevance, influence, or other properties of a message or product. Using these data, along with user demographic characteristics, the algorithms underlying the system generate personalized item recommendations for each user. As these systems learn more about the user, they can continually adapt to improve the recommendations.

Recommender systems can be implemented using 3 approaches: a content-based approach [25], a collaborative filtering approach [26], or a hybrid approach [27]. The distinction between a content-based approach and a collaborative filtering approach lies in the type of data used to generate a recommendation, as we discuss in further detail in the Results section. Hybrid approaches merge content and collaborative filtering [27]. By combining theory-based CTHC with the empirical approach of recommender systems, the hybrid approach is a potentially powerful combination.

The lower portion of Textbox 1 provides an example of how a recommender system could be implemented to provide tailoring as part of a smoking cessation intervention. It shows how applying a recommender approach to health promotion could potentially improve the tailoring provided by current rule-based CTHC approaches. The primary difference between current CTHC and recommender systems is how the content would be tailored. In current CTHC, study designers select the variables and develop the rules that specify how the content will be tailored. In recommender systems, machine learning algorithms use the data (patient profiles, and implicit and explicit feedback ratings) to select the variables and generate the rules that specify how the content will be tailored. As new data about the users are collected, these recommender systems have the ability to refine the variables and tailoring rules. Can we augment the performance of CTHC by using recommender systems?

We present information gained through a focused literature review and through interviews with subject experts. We begin with a description of the limitations of current CTHC systems. We then describe the potential advantages and challenges of using a recommender systems approach. Based on the evidence presented, we propose a future research agenda. Our goal is to promote discussion of techniques to improve current CTHC through use of recommender systems.

## Methods

### Study Design

We conducted a focused literature review and interviewed experts to explore whether and how recommender systems might enhance CTHC approaches. This study was conducted between October 2012 and September 2015.

### Data Collection

#### Literature Review

**Figure 1:**
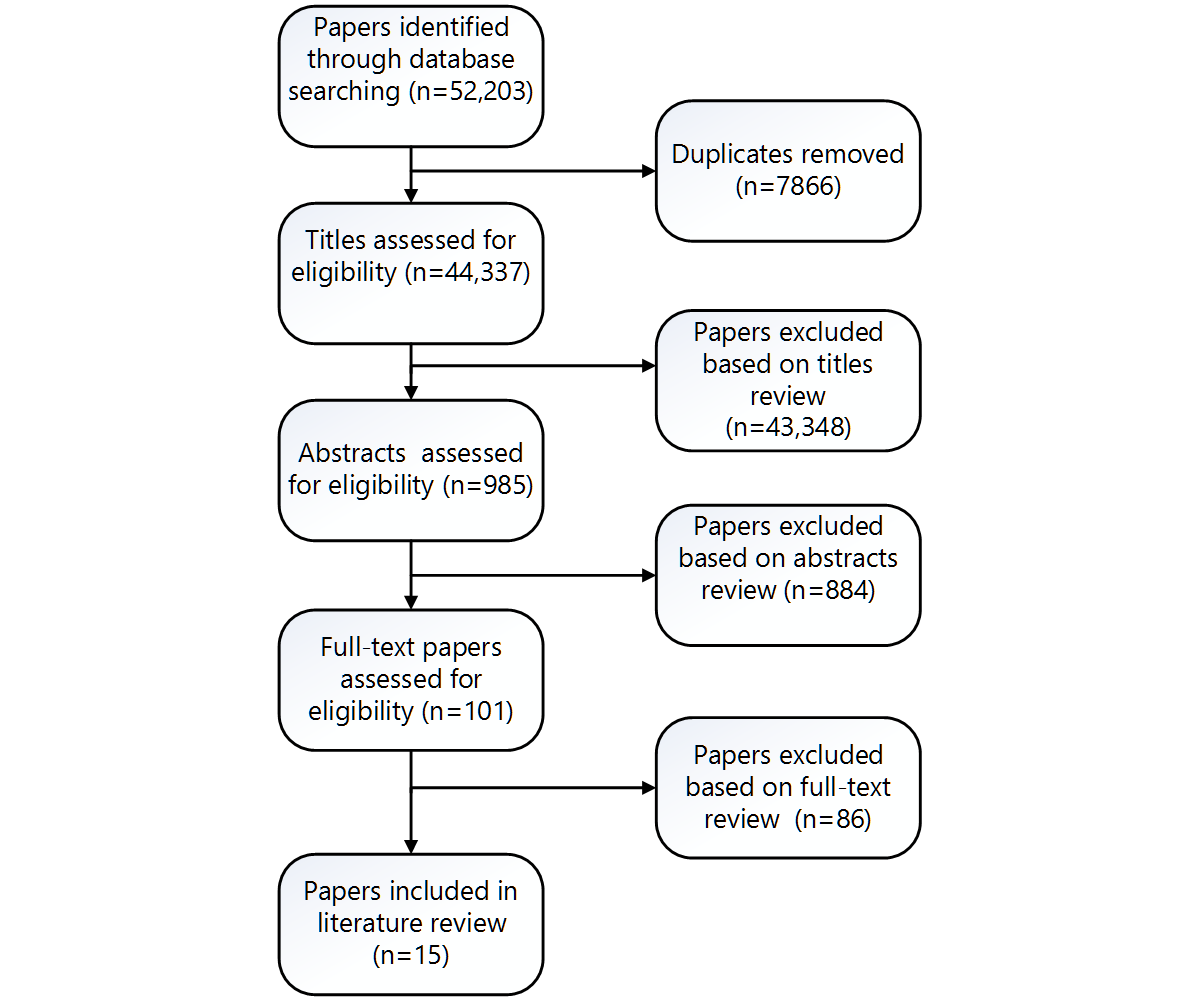
Literature review study flow diagram.

We conducted a focused literature review to identify white papers, conceptual papers, and peer-reviewed papers describing both current CTHC systems and recommender systems, as well as information for the following categories: limitations of current CTHC systems, advantages of recommender systems over rule-based systems, and challenges of implementing recommender systems for health promotion. We excluded papers that only described a specific intervention or a specific method for implementing these systems. Papers published in peer-reviewed journals and conferences between 1985 and 2015 from several disciplines, including clinical, health promotion, behavioral medicine, computer engineering, and recommender systems, were considered for the secondary literature review that was conducted from August 2015 through October 2015. The following databases were searched: PubMed, ACM Digital Library, and IEEE Xplore. Search terms for the Boolean search techniques were computer tailoring, health message tailoring, recommender systems, content-based, collaborative filtering, hybrid systems, and their combinations with health, overview, challenges, and barriers. Additionally, we reviewed the reference lists of all of the identified papers for additional relevant papers (Figure 1). Titles of the identified papers were reviewed by 1 reviewer (Kinney) under the supervision of Sadasivam. The number of papers identified in the initial search and included in the abstract review was vastly inflated due to variations in meaning of the search term (ie, hybrid systems referring to topics in electrical engineering). Papers that were excluded in the full-text review included those that were too speculative and opinion based, discussed only a specific trial or study, or were too similar to a paper that was already selected (eg, papers by the same authors). The team then synthesized the literature search results. Two authors (Sadasivam and Kinney) reviewed and summarized the information presented in the 15 papers under 3 overarching categories (limitations of current CTHC systems, advantages of recommender systems over rule-based systems, and challenges of implementing recommender systems for health promotion). These findings were presented to the coauthors for review and, as a group, we refined the points in each category.

#### Expert Interviews

We interviewed a purposive sample of experts in academia and at the National Institutes of Health (NIH) (n=8). We chose a sample size of 8 to assure representation in the 2 domains of interest (4 each): (1) computer engineering and recommender systems, and (2) health behavioral change, health communication, and computer tailoring. Interviewees were recruited through personal contacts and personal outreach at conferences, such as the Society of Behavioral Medicine, American Medical Informatics Association, and recommender systems annual conferences. We conducted individual interviews and used an open-ended interview format structured around the 3 themes: the limitations of current CTHC systems, potential advantages of recommender systems over rule-based systems, and challenges of implementing recommender systems for health promotion. In the beginning of the interview, the interviewer described the 2 types of systems (current CTHC and recommender systems) to promote discussion. Our literature findings organized around the 3 categories (limitations of current CTHC systems, advantages of recommender systems over rule-based systems, and challenges of implementing recommender systems for health promotion) were presented to the experts. We then used open-ended questions designed to solicit feedback from the experts around the 3 categories. Example questions were (1) Thinking about your last CTHC study, tell us how current CTHC systems limited your efforts in your study? (2) Thinking about your last CTHC study, tell us how you think recommender CTHC systems would have addressed current CTHC limitations? (3) What do you think are the challenges for using recommender systems in health interventions? Prompts were used when necessary. Example prompts were (1) Were you able to implement all the tailoring rules in your current CTHC study? (2) Do you think we have sufficient data to implement recommender systems? Detailed notes of each interview were taken. We used a process similar to the literature synthesis to summarize and extract information from the interviews. Specifically, the same 2 authors summarized key points and issues that were raised during the interviews (also organized into limitations of CTHC, potential advantages of recommender systems, and challenges) and presented these to the group for further synthesis.

## Results

We present the results of our data synthesis below.

### Limitations of Current CTHC

#### Source: Literature Review

**Figure 2:**
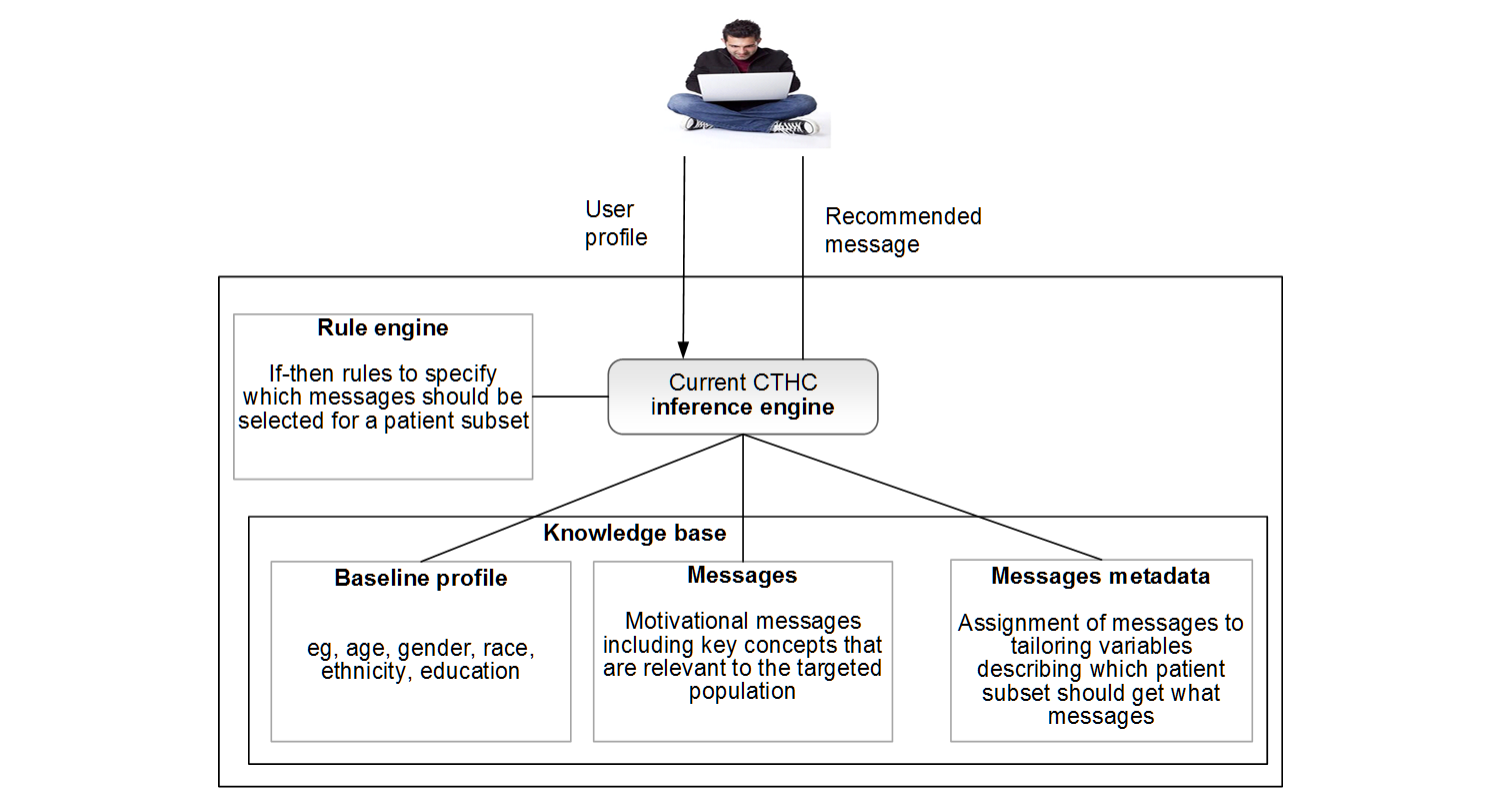
Structure of a current rule-based computer-tailored health communication (CTHC) system.

Current CTHC frameworks use theory-driven, rule-based systems to provide different messages to patient subsets [2,6,9,10,28-30]. Rule-based systems are one of the first and simplest forms of artificial intelligence, allowing automation of decisions in a manner consistent with rules provided by a human expert [31,32]. For example, rule-based systems are used as clinical decision-support systems to help providers choose the correct diagnosis. Conceptually, a rule-based system has 3 components: (1) a knowledge base that stores all facts from which the choice needs to be made, (2) rules that provide conditional statements that link the given conditions to facts, and (3) an inference engine that combines the rules and the knowledge base to suggest the optimal choices [31,32]. In developing these components (Figure 2), study designers must consider several questions [33-35]: (1) message writing: what are the important concepts for the targeted population? (2) tailoring variables: how should the target population be segmented? (3) rules: how should messages be selected for different segments of the targeted population?

In the Textbox 1 example, the tailoring variable was gender (male and female smokers), a key concept was addressing weight gain, and a rule was that half of the emails sent to women should contain information related to weight gain, but only a quarter of the emails sent to men should be focused on weight gain.

Once these questions are addressed and the messages written, study designers use metadata to describe and categorize the messages. This step allows the CTHC system to select appropriate messages for a patient subset. Metadata is defined as data about data; it describes the structure or content of a particular resource, object, or entity [36]. Previously applied to card catalogue systems within libraries, this concept is applied similarly in the electronic realm to discover concepts or resources. In a CTHC system, metadata are used to flag messages to help the inference engine identify which group of messages should be sent for a particular tailoring condition. In Textbox 1, examples of metadata include flagging for weight gain messages, and a gender flag to indicate whether the message should be sent to a man or woman, or there should be a common message for both genders. Thus, for John, the CTHC system would use the weight gain flag and the gender flag to select an appropriate message.

As study designers address the above questions and develop the intervention, they also have to balance several factors, including time and cost. This study designer-driven, rule-based approach may lead to 3 important limitations, detailed in the expert interviews.

#### Source: Expert Interviews

##### Tailoring on Multiple Variables is Challenging With a Rule-Based Approach

Leaders in the field of CTHC have demonstrated that high tailoring (tailoring on many variables) is better than low tailoring (using fewer variables) [37]. The use of rule-based expert systems may limit the complexity of CTHC systems in terms of the number of tailoring variables that can be included [2,6,9,10]. As noted, typically these rules are programmed using if-then statements [30,32,38]. The number of rules that can actually be implemented is dependent on several factors, including the programming team, the project’s budget, and the timeline. For example, if a smoking cessation CTHC system is tailored for gender (male, female) only, 2 if-then statements are required. By adding age (eg, 19-34, 35-44, ≥45 years) as a tailoring variable, the number of if-then statements required becomes 6. A third condition (smoking status: contemplation, preparation, and action) increases the number of if-then statements necessary to 18. In general, the number of patient segments increases exponentially as the number of tailoring variables increases. Thus, tailoring on many variables quickly becomes difficult to implement and very resource intensive.

##### A Theory-Based, Designer-Written Rules Approach May Limit Individual Relevance

While theory provides important guidance to CTHC investigators, current theories may underrepresent the complexity of factors that influence health behaviors [39,40]. This disconnect may be especially problematic when trying to reach diverse populations. Unique sociocultural dimensions, such as interconnectedness, level of health socialization, and ecological and health care system factors, influence personal perspectives and may affect the success of an outreach effort [28]. Testing of CTHC interventions in pilot studies may help to improve the interventions, but such pilots have limited effectiveness. Budgetary and time constraints often drive researchers to focus on improving messages for a few key concepts rather than capturing a broad perspective. In such situations, generalizability is limited and the risk of missing influential variables persists. The previously noted limitation on the number of rules that can easily be implemented also increases the possibility of key concepts being excluded.

##### Rule-Based CTHC Systems Often Have Limited Ability to Adapt in Real Time

A user’s personal preferences and behaviors can change over the duration of the intervention. An optimal CTHC system needs to have the capabilities to adapt in order to remain relevant and engaging. While the ability of current systems to collect real-time behavior has improved (eg, ecological momentary assessment and use of sensors), current CTHC rule-based approaches are limited in how they can adapt to this information. CTHC rule-based systems typically adapt only to anticipated and predicted changes in behavior (ie, how the study designers think users will behave). For example, current CTHC systems can be easily programmed to adapt to changes in a smoker’s motivation to quit. However, to adapt to all the behavioral patterns of the individual and the group, existing rules would need to be modified or new rules added. This approach quickly becomes resource intensive and often infeasible.

Using the Textbox 1 example, because the study designers did not choose weight gain as a tailoring variable and did not write rules for it, the rule-based CTHC was not able to personalize the intervention to John’s needs by sending him additional emails targeting weight gain. However, the recommender CTHC system was able to note John’s viewing of the weight gain page on the website (ie, implicit data) and then to send a message targeting weight gain to John early on in the intervention. Based on John’s ratings, the recommender system was able to further adapt and send additional messages targeting weight gain to him. Thus, John’s experience of the intervention was further enhanced because of the additional tailoring provided by the recommender system.

### Advantages of Incorporating Recommender Systems to Advance CTHC

#### Source: Literature Review

The use of complex algorithms to generate the tailoring recommendations based on collective-intelligence data allows tailoring based on the “observed behavior” of the users—how the users are responding to the intervention collected through user feedback, rather than how the study designers predict the users are going to respond. User feedback data can be in the form of explicit or implicit data. As noted, recommender systems can be implemented using 3 approaches: a content-based approach [25], a collaborative filtering approach [26], or a hybrid approach [27]. Content-based recommender systems use the description of the items (metadata) and the preferences of the user to make user-specific recommendations. Given a sample of rating data, content-based recommender systems learn a function to match users to items by taking the user profile information (eg, age, gender) and the metadata of the items as input. While content-based recommender systems conceptually work similarly to current CTHC systems, the main difference between them is that the matching function is specified by study designers in existing CTHC systems in the form of tailoring rules, while the matching function is optimized based on rating data in the case of content-based recommender systems. Since the matching function is learned from data and not specified by hand, it is feasible to consider many more demographic and tailoring variables.

In contrast to content-based recommender systems, collaborative filtering recommender systems match users to items by directly leveraging feedback ratings data (implicit or explicit) of the item (ie, messages in the case of CTHC). The simplest examples of this approach are nearest-neighbor methods [26]. These methods match a target user with other users who have given similar feedback ratings data regarding the items the users have rated in common. The set of users matched to the target user are referred to as the target user’s nearest neighbors. The method then recommends items to the target user that their neighboring users have rated highly. The assumption behind these methods is that if 2 users are observed to have close agreement on the feedback ratings of a sufficiently large number of items, they will likely agree closely on the ratings for the remainder of the items.

Hybrid recommender systems merge the strengths of content-based and collaborative filtering recommender systems [27]. These systems recommend items by merging information about user demographics and explicit item characteristics with information about how similar users rate items. This approach would technologically be the most challenging to develop, requiring an integrated framework that links together the different types of information into a unified model. Hybrid models can potentially bridge the world of theory-based approaches with empirical recommender systems-based approaches. For example, in the CTHC case it may become possible to first segment users according to a study designer-specified top-level tailoring variable such as smoking status, then using the recommender system algorithm to automatically and significantly refine the tailoring within each top-level segment based on all available information sources.

#### Source: Expert Interviews

**Figure 3:**
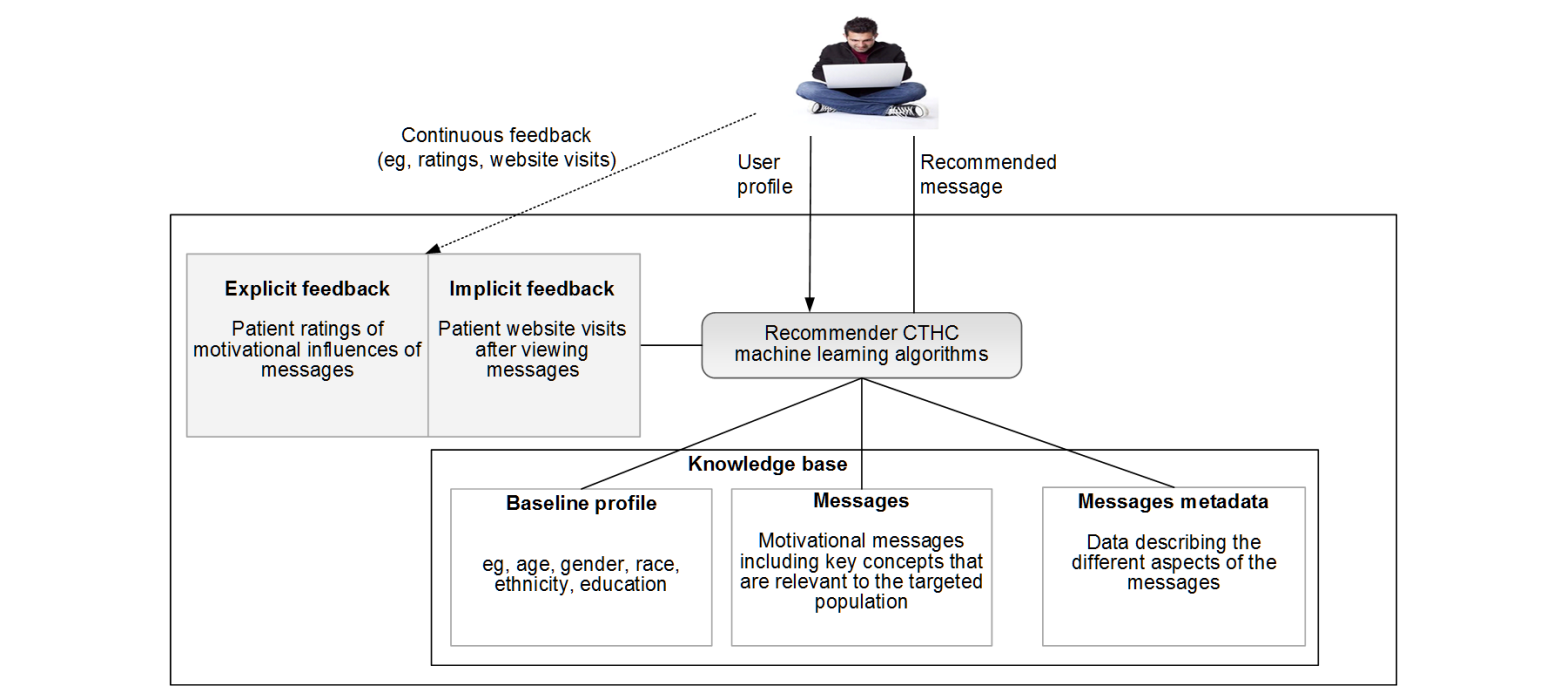
Components of recommender systems for computer tailoring. The primary differences between the 2 systems depicted in Figure 2 and 3 are shaded in gray. They represent the continuous feedback data that the recommender systems are able to use. CTHC: computer-tailored health communication.

When seeking to develop recommender system-based CTHC, study designers must face the following questions (see also Figure 3): (1) message writing: what are the important concepts for the targeted population? (2) tailoring variables: how do we collect the collective-intelligence data and what do we collect (including implicit and explicit data), and what data and variables can be made available for the algorithm in order to generate recommendations?

In the aforementioned smoking cessation example (Textbox 1), John’s rating of messages is explicit data, and his visiting a webpage on the website is implicit data. Along with John’s demographic characteristics, the recommender system was able to further personalize John’s experience on the intervention using these data.

As in current CTHC, in the recommender system study designers will have to develop metadata describing message characteristics that will be used for message selection. Study designers do not typically have to consider the selection of the tailoring variables and the rules, as these will be derived from the data collected by the algorithms underlying the recommender systems. This data-driven approach has the potential to provide several advantages. These are as follows.

##### Tailoring is Based on a Near-Infinite Number of Variables

Sophisticated machine learning algorithms are potentially able to consider all of the available user variables and to tailor based on these variables. As noted above, rule-based systems are limited in the number of variables that can be used. The recommender system approach potentially reduces the possibility of any key variable being excluded and allows for tailoring on more variables. The number of variables that can be effectively incorporated or is meaningful to the participant has to be empirically tested. Systems also have to be designed to collect all potential user data to take advantage of this ability of the recommender systems.

##### Tailoring is Not Limited to Theory or Study Designers’ Knowledge

A recommender systems approach would be an ideal complement to theory-based approaches because it would identify important variables from user data and behavior. The machine learning algorithms of recommender systems recommend messages based on the data and are not limited to the study designer-written rules. Textbox 1 provides an example of how recommender systems can augment theory-based approaches. By incorporating the smoker’s (John) feedback, the system was able to augment the tailoring provided by the system, sending additional messages targeting weight gain to the smoker.

##### Algorithms Can Adapt to Real-Time Feedback

In contrast to rule-based approaches, the machine learning algorithms of the recommender system can more easily adapt to unpredicted changes in individual as well as group user behavior. As noted in the Textbox 1 example, the system was able to note John’s browsing of a webpage on the site, as well as his ratings of the message, and used this information to further tailor the intervention. Thus, these systems can adapt to continuous feedback provided by the users of the intervention. Similar to the systems used by Netflix and Amazon, the system continually learns. As more users contribute data, more sophisticated message tailoring is possible.

Table 1 summarizes our above discussion on the differences between current CTHC and recommender systems, including how recommender systems can augment current CTHC. The next sections will describe potential challenges to incorporating collective-intelligence data and recommender systems in CTHC. In addition, we highlight important research questions.

**Table 1:** Rule-based computer-tailored health communication (CTHC) versus recommender systems.

Feature	Rule-based CTHC	Recommender systems
Intervention development questions	(1) Message writing: What are the important concepts for the targeted population?	(2) Message writing: What are the important concepts for the targeted population?
	(2) Tailoring variables: How should the target population be segmented?	(2) Tailoring variables: What collective-intelligence data (implicit and explicit data) should be collected and how?
	(3) Rules: How should messages for the participant patient segment be selected?	
Message selection	Rules-driven: Study designers develop rules based on the literature and theory. These rules link user profiles to the metadata of the messages, selecting messages for a patient subset.	Data-driven: Sophisticated machine learning algorithms derive the tailoring rules from the collective-intelligence data of the individual, as well as the group.
Complexity (number of variables)	The number of variables incorporated can become quickly unmanageable. It is limited by the sophistication of the study designers in the team, project’s timeline, and budget.	Sophisticated algorithms can potentially consider all the variables collected in the intervention.
Use of theory	Tailoring is limited to theoretical constructs.	Theory is augmented by deriving recommendations from the user data.
Adaptation	System is limited to predicted changes in behavior.	System can continuously adapt, potentially improving with each message delivered. Responds to the user’s behavior and to the group’s behavior over time.

### Challenges to Incorporating Recommender Systems in CTHC

#### Source: Expert Interviews

The potential is there, but can recommender systems be adapted to CTHC systems? There are several challenges or potential barriers to widespread adoption of this approach including.

##### Limited Availability of Collective-Intelligence Data at the Start of the Intervention

When companies such as Netflix and Amazon deployed their recommender systems, they had already collected collective-intelligence data on several thousands of users. In contrast, most CTHC interventions do not have access to such data sets, such as prior ratings of motivational messages, use and effectiveness data of an intervention, or sensor data from physiological measures of recipients’ reactions. The lack of such collective-intelligence data at the start affects the ability to reach and maintain sufficient momentum in the early stages of an intervention.

##### Limited Sample Size and Intervention Study Time

The sample size and study timeline of a typical behavioral health intervention impose additional challenges to a recommender system. In 2012, Amazon.com reported having a client base of over 100 million customers worldwide, while Netflix boasted 29.4 million users in that same year. In contrast, CTHC research settings draw on much smaller initial populations, often with limited user interaction. CTHC interventions have shorter timeframes, often due to the dictates of limited research funding. Small study populations and limited data collection may threaten both the generalizability of the messages and the precision of the algorithms.

##### Steep Rate of Intervention Attrition

Attrition rates tend to be very high in technology-assisted health interventions [41-46]. Typically, most users engage with the interventions only once or twice during the study. Combined with the limited sample size issue discussed above, this can significantly limit the availability of implicit feedback. Moreover, the users who engage more frequently may also be different from those users who are less engaged, and their feedback may not reflect the feedback of the less-engaged users [37,46].

##### Potential Unintended Consequence

There are potential unintended consequences of using a data-driven approach to tailor messages for users. Web 2.0 companies have developed over the years a sophisticated approach to collecting feedback data and channeling these data into their recommender systems. Explicit ratings in the form of “like” functions and implicit ratings, such as user webpage visits or purchase of a product, provide detailed ongoing feedback that informs subsequent messages sent to customers. While effectiveness of a message promoting online merchandise may be measured by users’ purchasing decisions, assessing the effectiveness of behavioral health messages is more complex. For example, users’ preferences could possibly tend toward information that reinforces the behavior that is being targeted for reduction. In other words, a user liking a message may not mean that the message will influence behavior change in the desired direction.

For example, triggers for smoking can vary among smokers [47]. In a hypothetical case, listening to music might be a useful relaxation technique for some participants, helping to reduce stress and to remove a trigger for smoking. However, for other smokers, listening to music might act as a trigger. A purely recommender system-based approach might not be able to distinguish between music as a relaxation device and music as a trigger. Avoiding unintended consequences of this type of situation would be challenging. Methods for monitoring the tailoring or for collecting data in ways that allow the system to make these distinctions would be important. Such approaches would need to be empirically tested.

## Discussion

### Proposed Research Agenda

#### Source: Expert Interviews

We propose the following research agenda to respond to the above challenges and to advance the field of CTHC using recommender systems approaches.

##### Research to Understand What Collective-Intelligence Data to Collect for Health Interventions and How

As noted, complicating the generation of collective-intelligence data is the lack of clarity of what constitutes appropriate feedback for health behavioral messages. Studies are needed to evaluate the research questions associated with this issue. We need to understand whether message feedback ratings on a single question (or dimension) are sufficient, or whether we need ratings on multiple questions. For example, a study designed to address this question could be to recruit users to rate messages on multiple dimensions (Textbox 2) and to assess whether these dimensions provide the same or different information. Because time and order of the questions could also be a factor in the ratings, the survey should be designed to present these questions in a random order. If the ratings of the messages are highly correlated, then having a single question might be sufficient. However, if they are not correlated, having additional questions might be useful. Researchers must balance the need for additional information with the burden of asking their users to rate multiple questions.

**Example dimensions of collective-intelligence data collection (further research might expand this list to include additional items).**

Motivational influence

This message influences me to change my behavior. (yes/no)

Emotional engagement

This message affected me emotionally. (positive and negative emotions)

Relevance

This message was personally relevant to me.

Preference

I would like more messages like this one.

Second, as noted above, using the wrong feedback data might lead to unintended consequences (see Results). Assessing whether a message might lead to unintended consequences could be challenging. One approach is to use technological advancements in data collection (eg, ecological momentary assessment or sensors) to assess the user’s behavior after receiving a message. For example, in a smoking cessation study, smokers who are attempting to quit could be provided with a mobile app to record any smoking and the reasons for smoking during the intervention. This information could then be compared with the messages that were sent immediately preceding the smoking event to assess whether that particular message was correlated with the smoking.

There are also a few strategies that can be studied to overcome the limited availability of collective-intelligence data. For example, the preintervention stage of a study can be used for explicit data collection. Research is necessary to determine the minimum amount of explicit data needed to develop a reasonably functioning CTHC algorithm**.** Research is also needed on how to continue gathering explicit data throughout the intervention. This could be in the form of a question at the end of every message sent to the participants. Research is also warranted on how to incorporate implicit data into the algorithm as the intervention participants engage with the system (eg, visits to a website).

Technological advances can also be used to generate additional collective-intelligence data. The considerable data warehouse technologies can be used to aggregate collective-intelligence data from multiple interventions. A new investigator can then use these data collected by other investigators to initiate this CTHC intervention. Another interesting development in recent years is the development of large social networks around health issues. For example, BecomeAnEx and QuitNet are social networks focused on helping smokers [48,49]. In these networks, users are continuously interacting with and supporting each other in their efforts to adopt health behaviors, while generating large quantities of untapped collective-intelligence data. One way to use these collective-intelligence data for CTHC would be to write natural language-processing tools to identify messages similar to an investigator’s health messages, and then mine the collective-intelligence data on these similar messages, such as the number of views of these messages and follow-up posts. This information could then be used to initialize a recommender system for a new intervention. Research is needed to examine whether these approaches are feasible and develop appropriate natural language-processing tools to extract information from existing social networks. Research is also needed to examine whether adding external data to the algorithms would be an improvement over cold starting [50] these systems without any data (ie, using rules to select the first set of messages and then personalize them based on user feedback) or with minimal data. A study could randomly assign participants to receive messages tailored either by a collective-intelligence system that is cold started or by a recommender system augmented by using data from other social networks. Users in the study could then be asked to rate each message that they receive. A comparison of the user’s ratings by time might provide insights into whether the collective-intelligence data enhanced the intervention. Questions include the following. Did the augmented collective-intelligence system receive higher ratings in the initial time periods as compared with the cold-started recommender system? Did this trend continue, or did the effect of augmenting with external data disappear after some time in the intervention?

##### Research to Understand Appropriate Selection of the Recommender Approach

As noted, recommender systems can be implemented using 3 approaches: content based, collaborative, or hybrid. Each of these has distinct advantages. While content-based systems are similar to rule-based approaches, content-based systems can use the rich metadata that can be developed for a particular message. While metadata is primarily used for flagging the messages to a particular tailoring condition, use of metadata in content-based systems can be more powerful. CTHC messaging can be described in several ways, including its relevance to particular concepts in a behavioral theory (eg, self-efficacy), the message polarity (positive, negative, or neutral sentiment), and the topical content of the message (eg, mentioning weight loss or cravings). In theory, a content-based system can use all this information in developing a matching function. However, in practice the cost of explicitly specifying large amounts of metadata for each message can be prohibitively expensive.

Collaborative filtering methods can bypass the need to match users to items based on explicitly defined metadata and instead derive recommendations based directly on items that similar users have rated highly. As a result, collaborative filtering recommender systems have been successful in domains such as book and movie recommendation, where enumerating all relevant characteristics of the users and items is difficult, if not impossible. However, there are certain disadvantages to using a purely collaborative filtering approach. This approach would imply that the tailoring is purely data driven and may lead to unintended consequences (see Results).

Hybrid systems can bridge theory-based, rule-based tailoring with the recommender empirical tailoring. While this might appear to be the best fit, it might not be feasible to develop hybrid models for all projects, given the limitations of time, content, and available collective-intelligence data. Thus, research is needed to identify the best recommender approach for an intervention and what approach would provide an advance over current rule-based approaches, make the intervention most engaging within the project constraints, and most influence the targeted behavior.

Studies are needed to compare the performance of all 3 approaches. For example, a study could directly compare the performance of all 3 approaches by randomly assigning participants to receive messages tailored by either a content-based, a collaborative, or a hybrid recommender system. Such a study could be evaluated in terms of several different outcomes. In a pilot study, the outcome could simply be a comparison of the ratings provided by participants for a period of time (eg, 30 days) or of the use of the intervention functions. Ratings could be in the form of explicit ratings (eg, Textbox 2 dimensions) or implicit ratings (eg, user webpage visits or setting a goal). In a long-term effectiveness study, the outcome could be desirable changes in the behavior (eg, quitting smoking). Investigators must also factor in the cost of developing these systems in their evaluations.

##### Research to Understand the Impact of Using Collective-Intelligence Data for CTHC

Will recommender systems be better than current CTHC? There is no evidence regarding the use of recommender systems in CTHC. Research is needed to understand the benefit of incorporating recommender approaches into CTHC, in terms of increased engagement as well as behavior change. Comparative effectiveness studies are needed to evaluate the relative impact of rule-based tailoring versus recommender systems tailoring across different health behavior targets. The outcome of such a study would be assessing the behavior change of interest, as well as increased engagement and satisfaction with the intervention.

To achieve these agenda, we may need changes in our training and funding models with an increased focus on supporting interdisciplinary research bridging behavioral science and computing. As with any interdisciplinary teams, researchers must be conscious of differences between disciplines in terms of terminology to ensure clear communication across team members. More fundamentally, researchers also need to be conscious of differences between disciplines in terms of where research challenges lie. For example, behavioral scientists may not be familiar with the challenges of developing, implementing, and deploying new algorithms and systems. On the other hand, computer scientists may not be familiar with the challenges involved in conducting behavior change intervention research, such as the time and effort needed to recruit subjects and ensure adequate levels of adherence to study protocols.

While this divide between disciplines has decreased with the increasing number of collaborations, additional training would speed up this merging. A model similar to the US National Science Foundation (NSF)/NIH mHealth Summer Institute training model might be a suitable approach to address some of these issues [51]. In the mHealth Summer Institute, behavioral scientists and mHealth researchers are brought together and exposed to the methodology and challenges faced by each respective field. Proposal development offers its own challenges. Investigators would benefit from attending conferences in the fields of those with whom they wish to collaborate, and the development of joint meetings may be beneficial in order to lay the groundwork for future proposal development.

As mentioned above, modifying existing funding models should also be considered. Developing recommender systems will require considerable time, which the typical NIH funding model does not facilitate. Substantial preintervention work will be needed to develop these systems, including collective-intelligence data collection through pilot surveys, recommendation algorithm development and validation, Web system design, message creation, and metadata creation. A joint NSF/NIH model similar to the Big Data Request for Applications that provides an additional development cycle and also stresses collaboration across disciplines might be a potential funding model for advancing the research agenda of using recommender systems in CTHC [52].

### Limitations

The views presented in this paper are limited. Research on the incorporation of recommender systems is in its infancy. Therefore, few papers relevant to this work have been published. We wrote this paper hoping it would start the conversation. Our hope is that the research community will consider the points presented in this paper and respond with additional issues that we have not yet considered.

### Conclusions

Recent technological advances and the widespread use of recommender systems outside health care present an incredible opportunity to improve on an already effective CTHC approach, and to reach and affect billions of users through Web and mobile technologies. Multiple challenges must be addressed to adapt recommender systems for CTHC. In this paper, we have attempted to start a discussion that we hope will help to move CTHC into the 21st century of these recommender systems.

## Abbreviations

CTHC: computer-tailored health communication
NIH: National Institutes of Health
NSF: National Science Foundation
